# Exploring the clinical outcomes and molecular characteristics of *Acinetobacter baumannii* bloodstream infections: a study of sequence types, capsular types, and drug resistance in China

**DOI:** 10.3389/fcimb.2025.1549940

**Published:** 2025-02-17

**Authors:** Jiao Chen, Yanting Shao, Zhibin Cheng, Guanghui Li, Fen Wan, Chenyan Gao, Danqin Wu, Dandan Wei, Yang Liu, Rong Li

**Affiliations:** ^1^ School of Laboratory Medicine, Nanchang Medical College, Nanchang, China; ^2^ School of Information Engineering, East China Jiaotong University, Nanchang, China; ^3^ Neurology Intensive Care Unit (ICU), First Affiliated Hospital of Nanchang University, Nanchang, China; ^4^ Department of Clinical Microbiology, the First Affiliated Hospital of Nanchang University, Nanchang, China; ^5^ Clinical Laboratory, China-Japan Friendship Jiang Xi Hospital, Nanchang, China; ^6^ Department of Clinical Laboratory & Jiangxi Province Key Laboratory of Immunology and Inflammation, Jiangxi Provincial People's Hospital & The first Affiliated Hospital of Nanchang Medical College, Nanchang, China

**Keywords:** *Acinetobacter baumannii*, bloodstream infection, carbapenem-resistant *Acinetobacter baumannii*, sequence type, capsular type

## Abstract

**Background:**

Bloodstream infections (BSIs) caused by *Acinetobacter baumannii* have been associated with high mortality. To improve the outcomes of patients, this study explored the clinical characteristics and outcomes of patients with BSIs, as well as the phenotypic and genomic characteristics of these isolates.

**Methods:**

A retrospective cohort study was conducted involving *A. baumannii* BSIs cases from 2020 to 2023 in a tertiary hospital. The clinical characteristics of all *A. baumannii* isolates were evaluated. Virulence phenotypes of all isolates were evaluated using the growth curve, biofilm-forming assay, antiserum complement killing, and *G.mellonella* killing assay. Furthermore, whole-genome sequencing (WGS) was utilized to analyze genomic characteristics.

**Results:**

The 30-day mortality rate of 67 patients with BSIs was 55.22%. Patients in the death group had significantly lower platelet counts and higher CRP levels than those in the survival group. Additionally, higher rates of antibiotic use (≥2 classes) and greater carbapenem exposure were observed. Among the isolates, CRAb accounted for 80.6%, ST2 accounted for 76.12%, and KL2/3/7/77/160 accounted for 65.67%. The predominant KL type was KL3, found in 19.4% of the isolates. All ST2 and KL2/3/7/77/160 isolates were CRAb. Among the isolates, 90.7% of the CRAb isolates coharbored *bla_OXA-23_* and *bla_OXA-66_*, while one coharbored *blaNDM-1* and *bla_OXA-23_*. Compared with non-ST2 and non KL2/3/7/ 77/160 infections, ST2 and KL2/3/7/77/160 infections had higher mortality rates (66.0% vs. 23.5%, P=0.002; 65.90% vs. 34.78%, P=0.015). Patients with ST2 and KL2/3/7/77/160 infections underwent more invasive procedures, received two or more antibiotics and carbapenem therapy before isolation, and had lower serum albumin levels. These isolates exhibited significantly higher resistance to antimicrobial agents. No significant differences in virulence phenotypes were observed between the two groups, except for biofilm formation between the ST2 and non-ST2 groups (P=0.035). However, these isolates harbored more virulence genes related to iron uptake and biofilm formation.

**Conclusion:**

The mortality rate associated with BSIs caused by *A. baumannii* is high. It is of great significance for clinicians to pay attention to the risk factors of the clinical characteristics of patients and to identify the ST and KL types of the strains causing the infection at an early stage.

## 1 Introduction

Over the past two decades, *Acinetobacter baumannii* has emerged as a significant pathogen of nosocomial infections, especially carbapenem-resistant *Acinetobacter baumannii* (CRAb) (Doi, 2019; Antimicrobial Resistance Collaborators, 2022). It poses a major global threat due to its ability to acquire a high level of antimicrobial resistance and tolerance to the environment (Cain and Hamidian, 2023; Cavallo et al., 2023). The incidence of CRAb has increased markedly in the last decade (Lasarte-Monterrubio et al., 2022; Potron et al., 2024). According to the Antimicrobial Resistance Surveillance Network (CHINET) program, the resistance rates of *A. baumannii* to meropenem and imipenem rose from 39.0% and 31.0% in 2015 to 73.7% and 73.4% in 2023, respectively (http://www.chinets.com/).

The mortality rate of infections caused by CRAb is high, particularly for BSIs (Paul et al., 2018; Wang et al., 2024). In recent years, the mortality rate of bacteremia caused by *A. baumannii* has been reported to be 42% to 69.4% (Yu et al., 2021; Falcone et al., 2023; Wang et al., 2024). Numerous factors contribute to the poor prognosis associated with *A. baumannii* bacteremia. Previous research has identified main risk factors, including mechanical ventilation, a higher age-adjusted Charlson comorbidity index, prolonged ICU stay, previous surgery, and thrombocytopenia, and certain virulence genes (Peng et al., 2021; Yu et al., 2021; Bai et al., 2022).

Analysis of the study of publicly accessible genome sequence data indicates that sequence type 2 (ST2) is the most predominant type (Hamidian and Nigro, 2019), which has been related to the poor clinical outcomes in *A. baumannii* bacteremia (Chuang et al., 2019).In addition, capsular polysaccharide (CPS) plays a critical role in the pathogenicity of *A. baumannii* (Geisinger et al., 2019; Morris et al., 2019). Different capsule types may influence antibiotic resistance, prognosis, and disease pattern (Hsieh et al., 2020).

In this research, we explored the clinical characteristics and outcomes of patients with BSIs, as well as the phenotypic and genomic characteristics of these isolates. Furthermore, we delineate the STs, capsule type distribution, and their associations with the prognosis in cases of with *A. baumannii* blood infection.

## 2 Methods

### 2.1 Patients and clinical data collection

Clinical data were collected from patients at the first affiliated University of Nanchang (Nanchang, China) with positive *A. baumannii* blood cultures between January1,2020 and March 31, 2023, using electronic health records. Data included patient demographics (age and sex), hospitalization period, invasive procedures, comorbid disease, other sites of *A. baumannii* isolation, laboratory examination, and antimicrobial usage, all analyzed retrospectively.

### 2.2 *A. baumannii* isolates

Identification of *A. baumannii* isolates was performed using matrix-assisted laser desorption/ionization mass spectrometry (bioMérieux, Marcy-l’Étoile, France) and PCR detection of *bla_OXA-51-like_*.Antimicrobial susceptibility testing was conducted with VITEK 2 Compact (bioMe´rieux) and interpreted according to the CLSI guidelines (CLSI, 2022). The minimal inhibitory concentrations of colistin and tigecycline were determined using the broth microdilution assay according to Clinical and Laboratory Standards Institute (2022) and U.S. Food and Drug Administration (FDA) standards, respectively (Zhang et al., 2022).

### 2.3 Growth curve

The single colony was inoculated into 5ml of Luria-Bertani broth and incubated at 37°C until it reached the logarithmic growth stage. The concentration of the bacterial solution was adjusted to 10^6^CFU/ml with LB broth, and 200μL of the bacterial solution was dispensed into 96-well plates, with an equal volume of broth used as a blank control. Incubation was performed at 120rpm and 37°C for 24 hours. The absorbance value (OD600) of the bacterial solution was measured every 30 minutes using a Multifunctional enzyme labeling instrument (Thermo Scientific, USA). Each experiment was conducted in triplicate to obtain an average value (Rakovitsky et al., 2023). The growth rates of isolates were calculated using the growthcurver R package.

### 2.4 Biofilm forming assay

Biofilm formation activity was assessed using the microtiter plate technique as described by Mahmoud AFK et al (Mahmoud et al., 2021). After overnight growth in LB broth at 37°C, isolates were adjusted to 0.5 McFarland standards and diluted 1:100 in LB broth. Then, 200μL of the adjusted bacterial cultures were incubated in 96-well polystyrene plates for 24 hours at 37°C. Following incubation, the wells were stained with 0.1% crystal violet for 20 minutes. After washing and allowing to dry naturally, the bound dye was dissolved in 200μL of ethanol, and absorbance was measured at 540 nm. Each strain was tested in triplicate, and the means of three repeated experiments were calculated.

### 2.5 Antiserum complement killing

To obtain sterile serum, blood was drawn from healthy participants, centrifuged, and filtered through a 0.22 µm pore syringe filter. By heating the serum for 30 minutes at 56°C in a water bath, half of the serum was rendered inactive. When *A. baumannii* reached 2 × 10^6^ CFU/ml during the logarithmic growth phase, it was combined with normal or inactivated serum at a 1:1 ratio. The samples were serially diluted and plated on MH agar plates after a one-hour incubation period at 37°C. Following an overnight incubation period at 37°C, the number of bacterial colonies was counted. The bacterial survival rate was calculated using the formula:(number of colonies in normal serum/number of colonies in inactivated serum) × 100% (Niu et al., 2020).

### 2.6 *In vivo G.mellonella* killing assay

Virulence *in vivo* was evaluated by a *G.mellonella* killing assay as previously described (Chen et al., 2023). The assay comprising of 10 larvae (250–350 mg; Huiyude, Tianjin, China) was performed using 1×10^6^CFU/mL *A. baumannii* strain for single infections, with PBS as a negative control for three times. The larvae were maintained at 37°C for 96 hours, with assessments for signs of death occurring every 24 hours. Larvae were considered dead when no movement was observed after gentle prodding with forceps. *A. baumannii* AB5075 and ATCC 19,606 were used as reference strains for high and low virulence, respectively.

### 2.7 Genomic analysis

All isolates were sequenced using the Illumina PE Cluster Kit (Illumina, USA). FastQC software was employed for quality control of the raw sequencing data. Clean data were assembled using SOAP Denovo 2.0. The raw sequencing data generated from this research have been deposited in NCBI SRA (http://www.ncbi.nlm.nih.gov/sra) under the accession number PRJNA1193765. Multilocus sequence typing (MLST) was performed using the Pasteur scheme, targeting seven housekeeping genes (cpn60,gltA, fusA,pyrG,recA,rpoB, and rplB) from the WGS sequences to determine Sts. The online tool MLST 2.0 was utilized. K locus (KL) types were determined using Kaptive version 2.0.6 (Wyres et al., 2022). Identification of antimicrobial resistance genes and virulence genes was achieved by the ResFinder database and the Virulence Factor Database (VFDB), respectively.

### 2.8 Statistical analysis

Statistical analysis was conducted using SPSS 25 and GraphPad Prism 8.0. Figures were produced using the R package and GraphPad Prism 8.0.

## 3 Results

### 3.1 MLST and KL types

Among the 67 strains, 14 distinct Sts and 20 distinct KL types were identified.ST2 was the most common, accounting for 76.12% of the strains.KL2/3/7/77/160 accounted for 65.67% of all *A.baumannii* isolates and 88.0% of CRAb. The predominant KL type was KL3, representing 23.6% for CRAb. All ST2 and KL2/3/7/77/160 isolates were CRAb.

### 3.2 Antimicrobial susceptibility

The isolates from the death group demonstrated significantly higher resistance to ceftazidime, cefepime, piperacillin-tazobactam, meropenem, imipenem, and ciprofloxacin than isolates from the survival group.ST2 isolates and KL2/3/7/77/160 isolates exhibited significantly markedly greater resistance to ceftazidime, cefepime, piperacillin-tazobactam, cefoperazone sulbactam, imipenem, meropenem, ciprofloxacin, levofloxacin, and tobramycin than non ST2 and non KL2/3/7/77/160 isolates. There was no significant difference in resistance to tigecycline, minocycline, or colistin between ST2 and non-KL2 isolates, KL2/3/7/77/160, and non KL2/3/7/77/160 isolates (**Table 1**).

**Table 1:** Comparison of the antimicrobial resistance rates of the isolates from the death and survival group, the ST2 and non ST2 isolates and the KL2/3/7/77/160 and non KL2/3/7/77/160 isolates.

Antimicrobial agents	Total (n=67)	Death (n=37)	Survival(n=30)	ST2 (n=50)	non ST2 (n=17)	KL2/3/7/77/160 (n=44)					non KL2/3/7/77/160(n=23)	*p*-Value (Death vs. Survive)	*p*-Value (ST2 vs. non ST2)	*p*-Value (KL2/3/7/77/160 vs. other KL types
						KL3(n=13)	KL77(n=9)	KL7(n=8)	KL2(n=7)	KL160(n=7)				
ceftazidime	79.10%	94.59%	63.33%	100.00%	17.65%	100.00%	100.00%	100.00%	100.00%	100.00%	39.13%	**0.001**	**<0.001**	**<0.001**
cefepime	82.09%	97.30%	66.67%	100.00%	29.41%	100.00%	100.00%	100.00%	100.00%	100.00%	47.83%	**<0.001**	**<0.001**	**<0.001**
piperacillin-tazobactam	80.60%	97.30%	60.00%	98.00%	29.41%	100.00%	100.00%	100.00%	100.00%	85.71%	47.83%	**<0.001**	**<0.001**	**<0.001**
cefoperazone sulbactam	53.73%	62.16%	43.33%	70.00%	5.88%	84.62%	55.56%	87.50%	57.14%	85.71%	17.39%	0.124	**<0.001**	**<0.001**
meropenem	80.60%	97.30%	60.00%	100.00%	23.53%	100.00%	100.00%	100.00%	100.00%	100.00%	43.48%	**<0.001**	**<0.001**	**<0.001**
imipenem	80.60%	97.30%	60.00%	100.00%	23.53%	100.00%	100.00%	100.00%	100.00%	100.00%	43.48%	**<0.001**	**<0.001**	**<0.001**
ciprofloxacin	79.10%	94.59%	60.00%	100.00%	17.65%	100.00%	100.00%	100.00%	100.00%	100.00%	39.13%	**0.001**	**<0.001**	**<0.001**
levofloxacin	62.69%	72.97%	50.00%	78.00%	17.65%	61.54%	88.89%	100.00%	100.00%	71.43%	26.09%	0.053	**<0.002**	**<0.001**
tobramycin	68.66%	75.68%	60.00%	90.00%	5.88%	84.62%	88.89%	100.00%	85.71%	85.71%	30.43%	0.169	**<0.001**	**<0.001**
sulfamethoxazole trimethoprim	67.16%	75.68%	53.33%	82.00%	29.41%	69.23%	77.78%	62.50%	100.00%	100.00%	43.48%	0.055	**<0.001**	0.451
minocycline	2.99%	5.41%	0.00%	4.00%	0.00%	0.00%	11.11%	0.00%	0.00%	14.29%	0.00%	0.196	0.402	0.299
tigecycline	0.00%	0.00%	0.00%	0.00%	0.00%	0.00%	0.00%	0.00%	0.00%	0.00%	0.00%			
colistin	0.00%	0.00%	0.00%	0.00%	0.00%	0.00%	0.00%	0.00%	0.00%	0.00%	0.00%			

Bold values are statistically significant (P < 0.05).

### 3.3 Clinical analysis according to the outcome of 30-day

A total of 67 patients with BSI caused by *A. baumannii* were included in the study. Thirty-seven patients died within 30 days, resulting in a mortality rate of 55.22%. Clinical data comparing the death and survival groups are presented in **Table 2**. The age and hospital stay duration of patients in the death group were significantly higher than those of the survival group (*P*<0.005, *P*<0.001), although the majority of patients in both groups were elderly and male. Most patients underwent invasive procedures, with tracheal intubation, mechanical ventilation, and Central venipuncture being the most common. The death group had a higher proportion of patients undergoing more invasive procedures involving mechanical ventilation and tracheal intubation compared to the survival group(*P*=0.029, *P*=0.015). Pulmonary infection was the most frequent comorbidity with *A. baumannii* isolated from the sputum of 34.33% of patients. The results of laboratory tests revealed that the death group had significantly lower platelet counts and higher C-reactive protein (CRP) levels than the survival group. Additionally, the death group had higher rates of antibiotic usage (defined as the use of two or more antibiotic classes) and greater exposure to carbapenems prior to blood separation (*P*=0.028, *P*<0.001)(**Table 2**).

**Table 2:** Clinical characteristics of patients with bloodstream infections caused by *Acinetobacter baumannii* according to the clinical outcome，ST type and KL type.

		Total (n=67)	survival (n=37)	death (n=30)	ST2 (n=50)	non-ST2 (n=17)	KL2/3/7/77/160 (n=44)					non KL2/3/7/77/160(n=23)	*p*-Value (Death vs. Survive)	*p*-Value (ST2 vs. non ST2)	*p*-Value (KL2/3/7/77/160 vs. non KL2/3/7/77/160 )
							KL3(n=13)	KL77(n=9)	KL7(n=8)	KL2(n=7)	KL160(n=7)				
Demographic															
	Age (years), mean±SD	57.21±17.51	50.63±18.50	62.54±14.88	58.84±16.50	52.41±19.97	62.46±13.53	62.67±10.77	68.63±17.40	55.00±21.06	50.00±15.481	51.00±19.00	**0.0048**	0.1933	**0.0348**
	Male gener number(%)	0.7164	76.67%	67.57%	72.00%	70.59%	61.54%	66.67%	87.50%	57.14%	71.43%	78.26%	0.206	0.911	0.385
Hospital stay days, mean±SD		28.9±24.74	40.83±29.86	19.22±13.74	30.72±27.07	23.53±15.45	30.85±33.12	24.33±27.87	34.38±26.65	29.86±27.05	37.43±25.15	24.78±17.55	**<0.001**	0.304	0.329
Hospital stay days bofore BSI , mean±SD		10.24±9.33	10.27±8.55	10.22±10.04	11.12±9.73	7.65±7.70	8.23±8.44	10.56±7.16	18.13±18.18	9.86±5.67	10.00±4.12	8.70±7.59	0.9826	0.187	0.332
Invasive procedures															
	Mechanical ventilation	43	15	28	37	6	8	7	7	6	6	9	**0.029**	**0.004**	**0.002**
	Central venous catheter	6	2	4	5	1	0	0	1	2	0	3	0.872	0.608	0.336
	Tracheal intubation	46	16	30	39	7	9	8	7	6	6	10	**0.015**	**0.005**	**0.001**
	tracheotomy	13	7	6	12	1	2	3	0	3	3	2	0.464	0.202	0.571
	Central venipuncture	41	15	26	37	4	10	7	7	4	6	7	0.09	**<0.001**	**<0.001**
	tracheoscope	4	1	3	3	1	1	1	1	0	0	1	0.763	1.000	1.000
	thoracentesis	14	6	8	12	2	2	1	3	1	3	4	0.871	0.202	1.000
	Abdominal puncture	12	5	7	11	1	4	1	1	1	2	3	0.811	0.140	1.000
Comorbid disease															
	Pulmonary infection	32	12	20	27	5	7	4	6	2	4	9	0.252	0.08	0.307
	Septic shock	4	2	2	2	2	0	1	0	1	0	2	1	0.565	0.890
	Respiratory failure	24	9	15	18	6	4	4	4	2	1	9	0.371	0.958	0.683
	Multiple organ failure	16	4	12	13	3	2	3	2	3	2	4	0.125	0.712	0.368
	hypertension	18	7	11	15	3	4	5	0	2	1	6	0.557	0.499	0.502
	diabetes	9	3	6	8	1	4	0	0	2	1	2	0.703	0.519	0.656
	hepatitis	9	5	4	6	3	3	0	1	0	2	3	0.735	0.859	1.000
	Coronary heart disease	4	1	3	3	1	1	1	0	0	0	2	0.763	1	0.890
A.baumannii isolated from other sites during hospital stay															
	sputum	23	10	13	20	3	7	3	2	2	5	4	0.877	0.167	**0.035**
	other sites	6	6	0	6	0	0	2	0	2	2	0	**0.006**	0.325	0.087
Laboratory examination,mean±SD															
	White blood cell count(×10^9^/L)	10.92±8.18	11.92±8.67	10.01±7.79	10.80±8.64	11.25±6.88	12.11±6.92	7.19±9.20	12.25±9.26	14.15±14.65	9.38±4.37	10.72±6.46	0.368	0.848	0.890
	Neutrophil count(×10^9^/L)	8.96±6.65	8.81±5.89	9.08±7.29	9.07±6.90	8.63±6.07	10.85±6.70	6.46±8.42	10.53±8.84	10.13±6.96	7.50±4.60	8.41±5.63	0.869	0.816	0.628
	Red blood cell count(×10^9^/L)	3.04±0.82	3.06±0.70	3.02±0.91	3.02±0.82	3.10±0.84	2.93±0.74	2.93±0.73	2.73±0.67	2.87±0.52	3.49±0.73	3.17±1.01	0.816	0.713	0.364
	Platelet count(×10^9^/L)	124.34±111.49	178.7±125.89	80.27±74.58	120.42±114.86	135.88±103.32	113.23±74.54	82.67±86.33	146.25±208.71	71.57±49.64	169.29±118.95	141.70±101.76	**<0.001**	0.625	0.361
	Albumin(g/L)	29.86±5.72	31.29±5.68	28.69±5.55	28.91±5.54	32.66±5.46	27.40±4.86	27.74±5.76	27.32±5.79	27.53±5.15	31.37±3.10	33.21±5.54	0.064	**0.018**	**<0.001**
	CRP (mg/L)	94.15±66.27	67.96±40.39	118.53±76.38	101.72±65.84	75.22±65.54	83.17±45.21	121.25±62.45	131.70±88.50	140.38±83.68	67.97±50.79	74.41±59.92	**0.003**	0.179	0.073
	Procalcitonin(ng/mL)	16.86±19.17	10.33±21.19	21.52±33.25	18.13±29.78	12.26±27.45	12.11±28.47	15.82±26.77	8.41±14.95	33.17±33.98	7.61±7.94	20.38±36.19	0.144	0.525	0.529
	Leukopenia(<4×10^9^/L)	12	3	9	10	2	1	4	2	1	0	4	0.23	0.69	0.936
	Agranulocytosis(<0.5×10^9^/L)	5	1	4	5	0	0	4	1	0	0	0	0.49	0.219	0.112
Usage of antimicrobials,n(%)															
	The numbers of antibiotic classes used before isolating from blood≥2	41	15	26	38	3	7	8	6	7	6	7	**0.028**	**<0.001**	**<0.001**
	Carbapenem therapy before isolating from blood	34	7	27	31	3	7	8	4	6	4	6	**<0.001**	**0.002**	**0.002**
Outcome															
	30-day mortality (%)	55.22%			66.00%	23.53%	84.62%	77.78%	37.50%	71.43%	42.86%	34.78%		**0.002**	**0.015**

Bold values are statistically significant (P < 0.05).

### 3.4 Clinical analysis according to ST and KL types

In terms of clinical analysis based on ST and KL types, the 30-day mortality rate among patients with ST2 and KL2/3/7/77/160 infections was higher than that among patients with non-ST and non KL2/3/7/77/160 infection (*P*=0.002, *P*=0.015). Compared to patients with non-ST2 infection, patients with ST2 isolates underwent more invasive procedures, including mechanical ventilation, tracheal intubation, and central venipuncture(*P*=0.004, *P*=0.005, *P*<0.001). They were also given two or more antibiotics and received carbapenem therapy before blood isolation (*P*<0.001, *P*=0.002), and had lower serum albumin levels (*P*=0.018). Similarly, compared to patients with non-KL2/3/7/77/160 infections, a greater percentage of patients in the KL2/3/7/77/160 group underwent invasive procedures of mechanical ventilation, tracheal intubation and central venipuncture (*P*=0.002, *P*=0.001, *P*<0.001), and were given two or more antibiotics and carbapenem therapy prior to blood isolation (*P*=0.002, *P*=0.015), while also exhibiting lower serum albumin levels (*P*<0.001, *P*=0.002). Furthermore, a higher incidence of *A. baumannii* was isolated from sputum samples during hospitalization (*P*=0.035) (**Table 2**).

### 3.5 Phenotype analysis of virulence in *A. baumannii*

**Figure 1:**
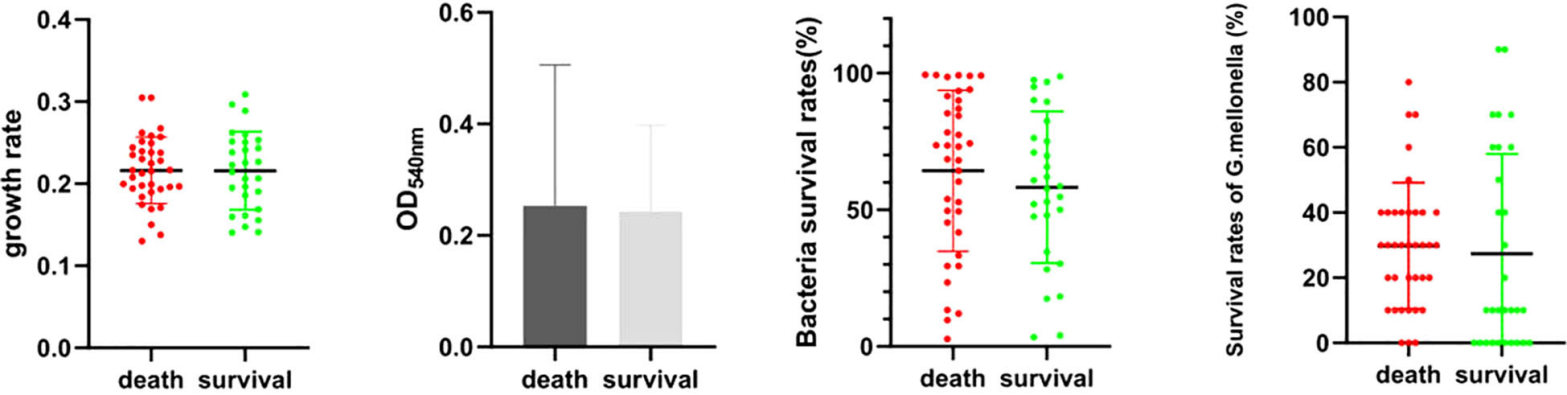
The virulence of isolates from the death and survival group (the growth curve, antiserum complement killing, biofilm-forming, and *G. mellonella* killing assay *in vivo*).

**Figure 2:**
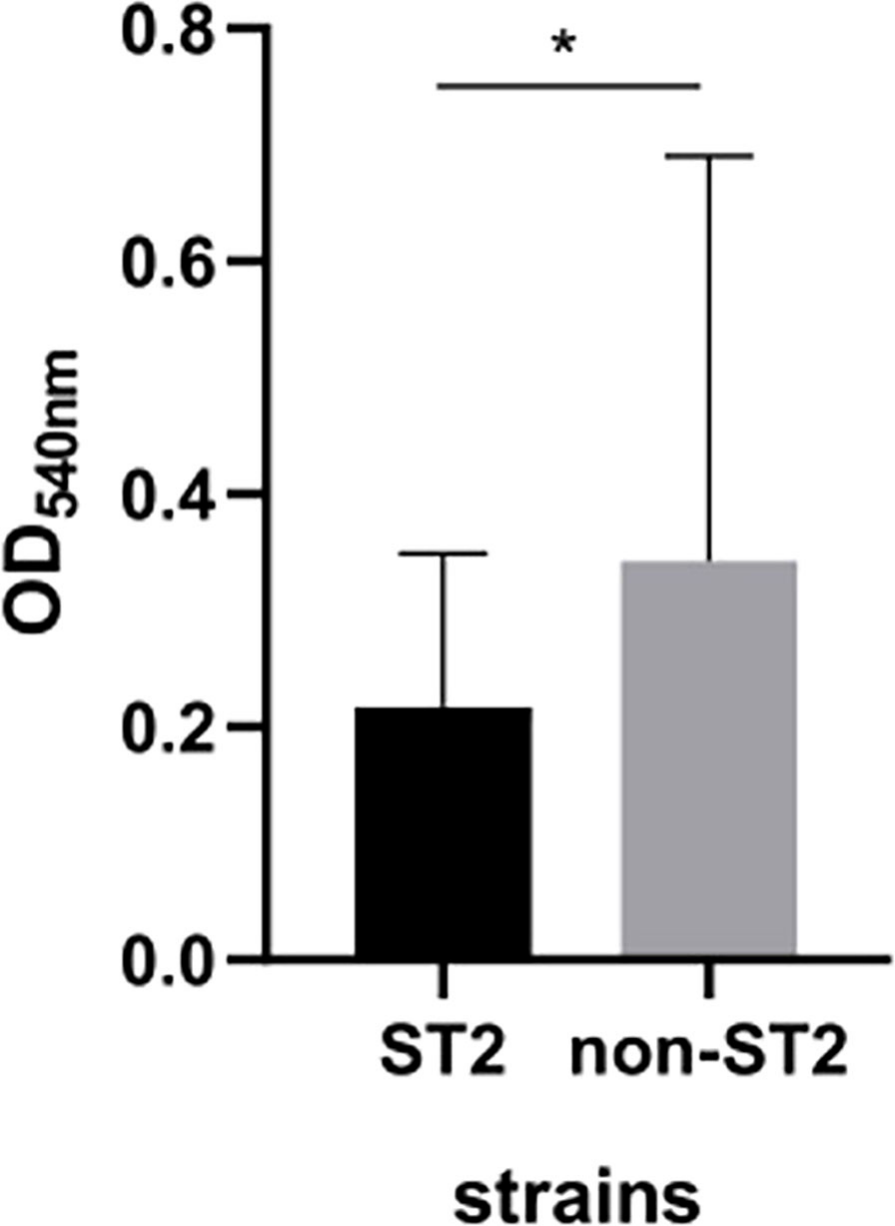
Biofilm formation ability of ST2 and non ST2 isolates. * *p* <0.05.

**Figure 3:**
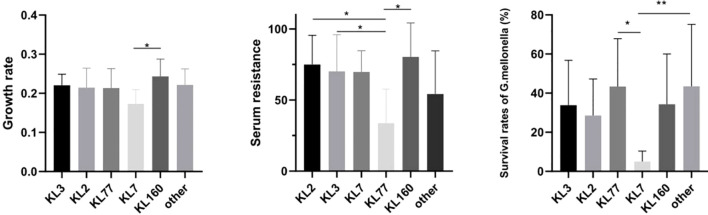
The virulence of KL2/3/7/77/160 and non KL2/3/7/77/160 isolates ( the growth curve, antiserum complement killing, and *G. mellonella* killing assay *in vivo*). *p <0.05, **p <0.01.

In our study, we assessed the virulence of all isolates using the growth curve, antiserum complement killing, biofilm-forming, and *G. mellonella* killing assay *in vivo*. The growth curve, antiserum complement killing, biofilm-forming, and *G. mellonella* killing assay *in vivo* were not statistically different between the survival and death groups (*P*=0.956, *P*=0.843, *P*=0.397, *P*=0.697) (**Figure 1**). There were also no significant differences in growth curves, antiserum complement killing, and *G. mellonella* killing assays between the ST2 and non-ST groups (*P*=0.911, *P*=0.423, *P*=0.118). The biofilm-forming ability of non-ST2 isolates was significantly stronger than that of ST2 isolates (*P*<0.05) (**Figure 2**). While there were no differences in virulence phenotypes between KL2/3/7/77/160 and non KL2/3/7/77/160, differences were observed among various KL types. Specifically, the growth rate of KL7 was slower than that of KL160 (*P*=0.026), and serum resistance in KL77 was lower than that in KL2, KL3, and KL160 (*P*=0.027, *P*=0.021, *P*=0.008). Furthermore, the survival rates of *G. mellonella* in KL7 isolates were lower than those in KL77 and other KL isolates (*P*=0.033, *P*=0.006) (**Figure 3**).

### 3.6 Genome analysis of *A. baumannii* based on WGS

**Figure 4:**
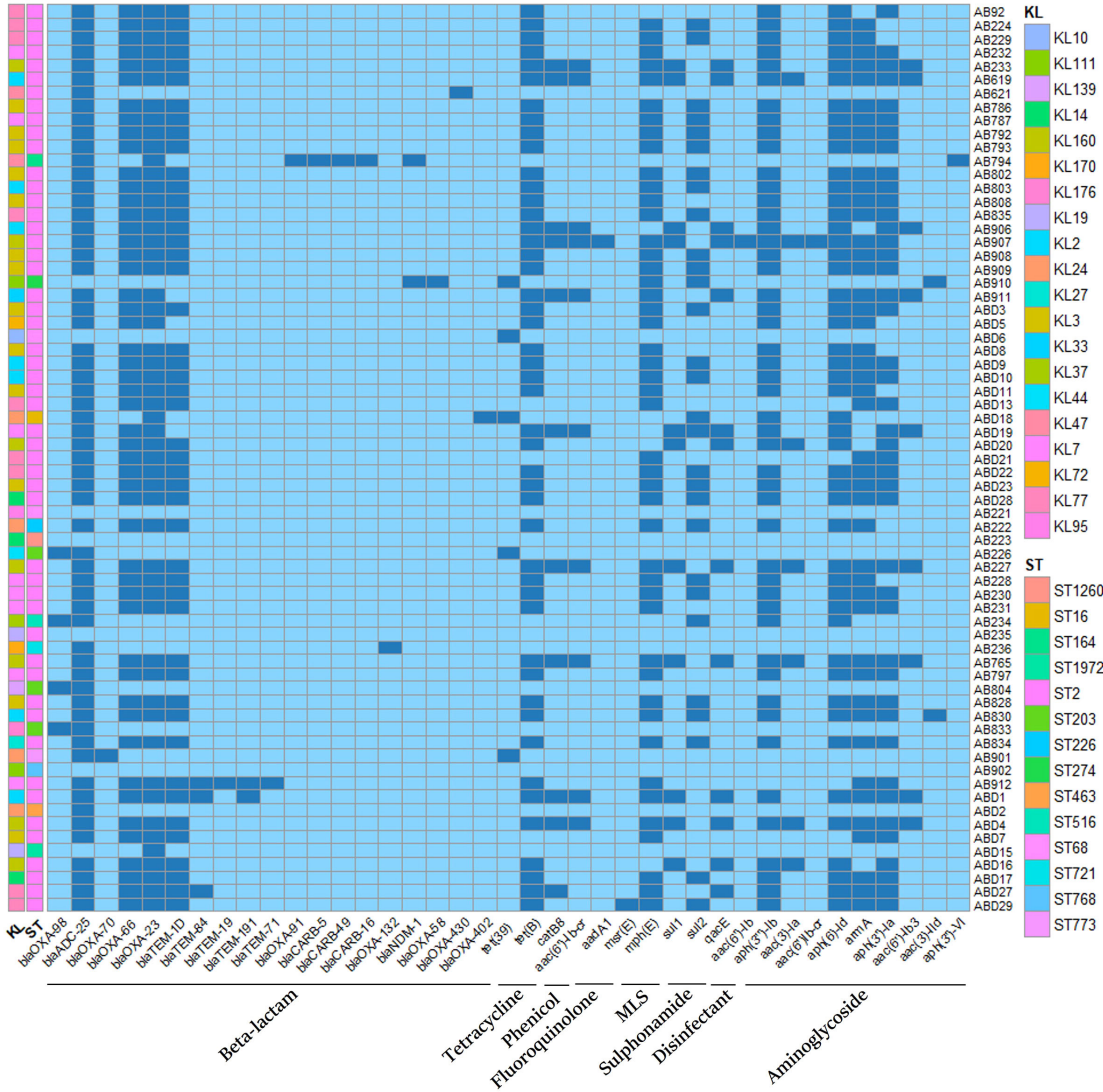
Drug-resistant gene analysis of all isolates in this study. STs and KL types are included. The presence of genes is in dark blue, and the absence of genes is in light blue. MLS, Macrolide, Lincosamide and Streptogramin B.

**Figure 5:**
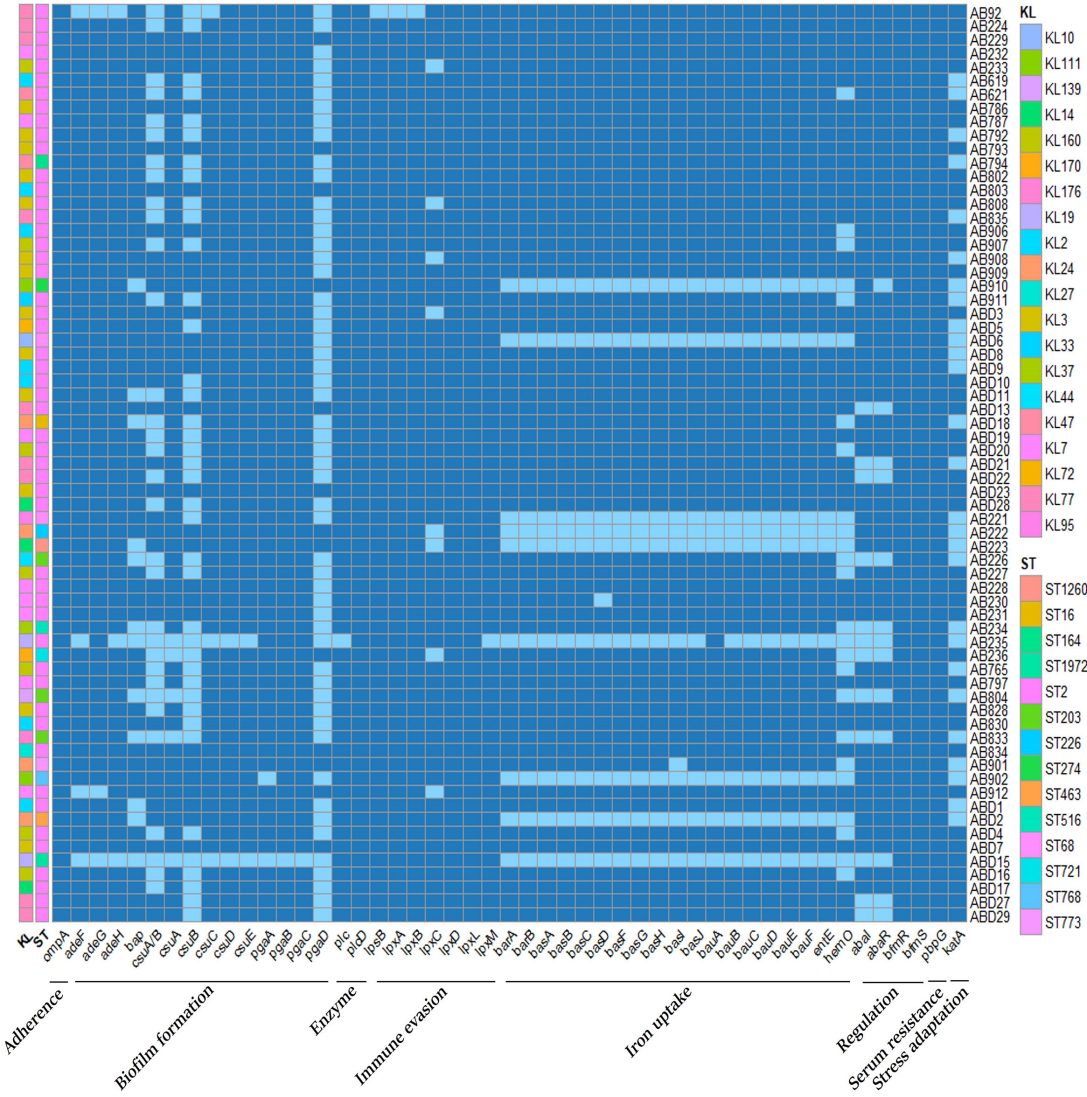
Virulence gene analysis of all isolates. STs and KL types are included. The presence of genes is in dark blue, and the absence of genes is in white.

In our study, all CRAb isolates harbored carbapenemase genes, including *bla_OXA-23_* (52/54), *bla_OXA-66_* (49/54), *bla_NDM-1_* (2/54), *bla_OXA-91_*, *bla_OXA-132_*, *bla_OXA-430_*.Notably, 90.7% of the CRAb isolates(49/54)co-harbored *bla_OXA-23_* and *bla_OXA-66_*, while one isolate co-harbored *bla_NDM-1_* and *bla_OXA-23_*. The antimicrobial resistance genes of all isolates are presented in **Figure 4**. Analysis of virulence genes revealed that a total of 49 virulence genes (ompA, adeFGH, bap, csuA/BABCDE, pgaABCD, plc, plcD, lpsB, lpxABCDLM, barAB, basABCDFGHIJ,bauABCDEF,entE,hemO,abaIR,bfmRS,pbpG, and katA). These genes are involved in multiple virulent functions, including serum resistance, biofilm formation, adhesion, iron uptake, etc. Strains isolated from the death group exhibited significantly higher levels of iron uptake (barAB, basABCDFGHIJ, bauABCDEF,entE, hemO), Biofilm formation (bap, csuA), Regulation (abaI, abaR) genes compared to those from the survival group (*P*<0.05). ST2 strains harbored more of Iron uptake (barAB, basABCDFGHIJ, bauABCDEF, entE, hemO), Biofilm formation (bap,csuA), Regulation (abaI, abaR) and Stress adaptation (katA) genes than those from the non-ST2 strains (*P*<0.001). Moreover, KL2/3/7/77/160 isolates exhibited increased levels of iron uptake (barAB, basABCDFGHIJ,bauABCDEF,entE, hemO), Biofilm formation (bap,csuA), and Stress adaptation (katA) genes compared to non KL2/3/7/77/160 (*P*<0.001). The virulence genes of all isolates are shown in **Figure 5**.

## 4 Discussion

*A. baumannii* bloodstream infections pose a serious hazard to human health due to their high mortality rates (Gu et al., 2022; Hassoun-Kheir et al., 2024). In our study involving 67 BSI patients, the 30-day mortality was 55.2%. Our findings indicate that factors such as age, CRP levels, the frequency of invasive procedures (mechanical ventilation and tracheal intubation), antimicrobial resistance, the number of antibiotic classes used (≥2), and exposure to carbapenems were significantly higher in patients from the death group compared to those in the survival group. Consistent with previous studies, mechanical ventilation and prior antibiotic exposure were identified as risk factors for BSI patients (Zhong et al., 2022; Zhang et al., 2023).

A nationwide retrospective research conducted across 19 hospitals in Italy analyzed the mortality associated with bloodstream infections caused by carbapenem-resistant Gram-negative bacilli, revealing that carbapenem resistance significantly increases mortality rates in BSI patients (Falcone et al., 2023). In our study, 80.6% of the 67 patients with *A. baumannii* bloodstream infections were identified as CRAb (54/67). Once *Acinetobacter baumannii* develops resistance to carbapenems, it often exhibits resistance to most other antimicrobial agents, resulting in limited treatment options. Currently, there is no clear “standard of care” antimicrobial regimen for CRAb infections. Moreover, there is insufficient data to demonstrate the additive benefits of routinely used combination regimens or the prioritization of specific agents with CRAb activity (Tamma et al., 2024). The primary treatment recommendations for extensively drug-resistant *A. baumannii* in China include tigecycline-based regimens, polymyxin-based regimens, and sulbactam-based regimens (Guan et al., 2016).In our study, 73.0% (27/37) of patients in the mortality group and 43.3% (13/30) of patients in the survival group received these regimens. Notably, 54.1% (20/37) of patients in the mortality group underwent changes in their anti-infective treatment regimen (two or more alterations). Studies have indicated that early and rational antibiotic use can reduce mortality of *A. baumannii* infection (Erbay et al., 2009). The lack of standardized treatment in this study might have contributed to the high mortality rate observed. More data are necessary to validate a rational antimicrobial regimen for treating CRAb infections.

Studies on the molecular epidemiology of global CRAb have shown that *bla_OXA-23_* and *bla_OXA-24/40_* are the most frequently acquired carbapenemase genes (Wang et al., 2024).In our study, carbapenem resistance of all CRAb isolates was mediated by carbapenemase genes, with 96.3% (52/54) of CRAb isolates carrying *bla_OXA-23_*, 90.7% (49/54) carrying *bla_OXA-66_* and 3.7% (2/54) carrying *bla_NDM-1_*. Notably, *bla_OXA-23_* remains globally predominant. Among the two strains producing NDM-1, one isolated in July 2022 co-harbored OXA-23, while *A. baumannii* was not isolated from the patient’s sputum or stool. The other strain, isolated in January 2023, came from a patient colonized in the respiratory tract with *A. baumannii*. However, the drug-resistance phenotypes of the two strains differed. The strain co-producing OXA-23 and NDM-1 (ST2; KL47) was different from reported strains (ST2; KL9) (Martins-Gonçalves et al., 2024), suggesting potential contamination from the hospital environment, for example through invasive procedures, et al (Miltgen et al., 2021).

Simultaneously, we evaluated differences in phenotypes and genotypes of virulence between the survival and the death group. No differences were observed in virulence phenotypes (growth rate, serum resistance rate, biofilm formation, and survival rates of *G. mellonella*) between the groups. However, strains from the death group harbored more Iron uptake (barAB, basABCDFGHIJ, bauABCDEF, entE, hemO), Biofilm formation (bap, csuA), and Regulation (abaI, abaR) genes than those from the survival group. Based on large and representative bacterial genomic data, a study indicated that the presence of the additional haem-uptake system hemO may have contributed to the success of certain *A. baumannii* clones, as the expression of several iron-uptake clusters has been connected to virulence (Artuso et al., 2023). The *bap* gene generates certain proteins at the cell surface that are directly related to biofilm formation and bacterial transmissibility. *cusA/BABCDE* is engaged in the first surface attachment during biofilm formation (Rezania et al., 2022).

Although new lineages are currently widespread in certain regions, the expansion of two major clones, GC1 and GC2, has been significantly responsible for the global spread of CRAb, especially GC2 (Wang et al., 2024).ST2 as the representative ST type of GC2 has been identified as the predominant MLST of *A. baumannii* (Liu et al., 2022; Duan et al., 2024) and was related to severe infection, inappropriate antibiotic treatment, and poor clinical outcomes in BSI (Chuang et al., 2019). Our study confirmed that the mortality rate for patients with ST2 isolates was significantly higher than for those with non-ST2 isolates (66.0% vs. 23.5%; *P* = 0.002). Patients with ST2 isolates underwent more invasive procedures, received two or more antibiotics, and carbapenem therapy prior to blood isolation, and exhibited lower serum albumin levels. The ST2 strains harbored more iron uptake, biofilm formation (bap, csuA), regulatory (abaI, abaR), and stress adaptation (katA) genes than non-ST2 strains. Nonetheless, there were no differences in virulence phenotype tests (growth rate, serum resistance rate, and survival rates of *G. mellonella*). Interestingly, non-ST2 isolates exhibited stronger biofilm formation ability, potentially due to differences in gene expression or other mechanisms.

In the genome of *A. baumannii*, capsule type is mainly determined by the genes responsible for CPS biosynthesis at the K locus (KL) of the chromosome (Kenyon and Hall, 2013). At present, over two hundred distinct K loci genes have been identified (Kenyon and Hall, 2021; Cahill et al., 2022). A study by Kelly L et al. analyzed 2,944 *A. baumannii* genomes and identified the most common KL types as KL2 (24.2%), KL9 (11.6%), KL22 (11.3%), KL3 (10.0%), and KL13 (5.3%) (Wyres et al., 2022). Wang et al. reported the most prevalent KL types as KL22, KL2, KL125, and KL3 among 842 CRAb isolates from various global regions (Wang et al., 2024). Yu-Chia Hsieh et al. screened K-types in large numbers of *A. baumannii* bloodstream isolates using the wzy-PCR combined with wzc-based method, finding KL2, KL10, KL22, and KL52 to be the prevalent capsular types of CRAb, patients with KL2/10/22/52 infection had a higher 30-day mortality rate (Hsieh et al., 2020). Our study is one of only a few studies that describe the epidemiology of the KL types of *A. baumannii* bloodstream infections through WGS and analyze virulence characteristics. We found that KL2/3/7/77/160 were the most prevalent KL types with KL3(24.1%), KL77(16.7%), KL7(14.8%), KL2(13.0%), KL160(13.0%), accounting for 88.0% of CRAb totally. Among them, KL7 *A.baumannii* isolates were described in the mucoid *A. baumannii* previously as highly virulent strains in *G.mellonella* survival assay (Chen et al., 2024). A study of tigecycline-resistant *A. baumannii* genome analysis indicated the KL7 cluster carried more antimicrobial resistance genes than other clusters (Qian et al., 2024). Interestingly, Rare studies have been reported on KL77 and KL160, suggesting the potential emergence of new clones in Nanchang, China.

Compared to patients with non-KL2/3/7/77/160 infections, the thirty-day mortality rate of the patients with KL2/3/7/77/160 infection is higher (65.90%vs 34.78%; *P* = 0.015). Notably, the mortality rate of KL3 was 84.62% (11/13).KL2/3/7/77/160 infections were more frequently associated with older age, invasive procedures, use of two or more antibiotics, prior exposure to carbapenems before blood isolation, and lower serum albumin levels. We analyzed the resistance phenotypes, virulence phenotypes, and genomic characteristics of both groups and found that KL2/3/7/77/160 strains had a higher resistance rate to other antimicrobials, except tigecycline and polymyxin. Additionally, these strains harbored more genes related to iron uptake, biofilm formation, and stress adaptation than the non-KL2/3/7/77/160 group. However, no significant difference in virulence phenotype was observed between KL2/3/7/77/160 and non-KL2/3/7/77/160 types, despite variations among the different KL types. Considering the limitations of our study, further research is needed to assess the impact of *A. baumannii* BSI with different KL types on patient outcomes.

Jia-Ling Yang et al. demonstrated that KL2 *A. baumannii* is associated with higher antimicrobial resistance and increased mortality from bacteremia (Yang et al., 2022). A study by Yu-Chia Hsieh et al. found that patients with KL2/10/22/52 infections exhibited higher rates of pneumonia, APACHE II scores, intensive care unit admissions, and Pitt bacteremia scores compared to those with non-KL2/10/22/52 infections. Additionally, patients with KL2/10/22/52 infections had a higher cumulative incidence of 30-day mortality (Hsieh et al., 2020). Identifying KL types may be crucial for understanding the virulence of *A. baumannii* and predicting outcomes in BSI. This necessitates the inclusion of more strains from other hospitals in epidemiological research to identify which KL types of *A. baumannii* are more likely to result in poor prognoses, along with further analyses of virulence and drug resistance characteristics.

In conclusion, the mortality rate of BSI caused by *A. baumannii* is high. Hospitals should enhance the management of *A. baumannii* infections. The prognosis of BSI caused by *A. baumannii* is influenced by numerous factors. It is of great significance to focus on the clinical characteristics of patients and to identify the ST and KL types of the strains causing the infection at an early stage.

## Data Availability

The datasets presented in this study can be found in online repositories. The names of the repository/repositories and accession number(s) can be found in the article/supplementary material.
