# Corrigendum: Exploring the clinical outcomes and molecular characteristics of *Acinetobacter baumannii* bloodstream infections: a study of sequence types, capsular types, and drug resistance in China

**DOI:** 10.3389/fcimb.2025.1585728

**Published:** 2025-03-19

**Authors:** Jiao Chen, Yanting Shao, Zhibin Cheng, Guanghui Li, Fen Wan, Chenyan Gao, Danqin Wu, Dandan Wei, Yang Liu, Rong Li

**Affiliations:** ^1^ School of Laboratory Medicine, Nanchang Medical College, Nanchang, China; ^2^ School of Information Engineering, East China Jiaotong University, Nanchang, China; ^3^ Neurology Intensive Care Unit (ICU), First Affiliated Hospital of Nanchang University, Nanchang, China; ^4^ Department of Clinical Microbiology, the First Affiliated Hospital of Nanchang University, Nanchang, China; ^5^ Clinical Laboratory, China-Japan Friendship Jiang Xi Hospital, Nanchang, China; ^6^ Department of Clinical Laboratory & Jiangxi Province Key Laboratory of Immunology and Inflammation, Jiangxi Provincial People’s Hospital & The first Affiliated Hospital of Nanchang Medical College, Nanchang, China

**Keywords:** *Acinetobacter baumannii*, bloodstream infection, carbapenem-resistant *Acinetobacter baumannii*, sequence type, capsular type

In the published article, there was an error in the **Funding** statement. The original text:

“The author(s) declare financial support was received for the research, authorship, and/or publication of this article. This research was financially supported by the National Natural Science Foundation of China (82200194, 82102411,32370195,62362034), the Project of Science and Technology Innovation Talents in Jiangxi (JXSQ2019201102), the Clinical Research Nurture Project of the First Affiliated Hospital of Nanchang University (YFYLCYJPY202001), Natural Science Foundation of Jiangxi Province of China (20232ACB202010), the Jiangxi Provincial Health Commission-Health Commission science and technology project (SKJP220226762, 202310458), and Institutional Research Project of Nanchang medical college (NYXJ-2024-040).”

The correct **Funding** statement appears below.

“The author(s) declare financial support was received for the research, authorship, and/or publication of this article. This research was financially supported by the National Natural Science Foundation of China (82200194, 82102411,32370195,62362034), the Project of Science and Technology Innovation Talents in Jiangxi (JXSQ2019201102), the Clinical Research Nurture Project of the First Affiliated Hospital of Nanchang University (YFYLCYJPY202001), Natural Science Foundation of Jiangxi Province of China (20232ACB202010), the Jiangxi Provincial Health Commission-Health Commission science and technology project (SKJP220226762, 202310458), Project of Innovation Team on Inflammation and Cellular Immunity, and Institutional Research Project of Nanchang medical college (NYXJ-2024-040).”

In the published article, there was an error in the **Abstract**, *Results*. The P-value of biofilm formation between the ST2 and non-ST2 groups is incorrect. It should be 0.035.

This section previously stated:

“Results: The 30-day mortality rate of 67 patients with BSIs was 55.22%. Patients in the death group had significantly lower platelet counts and higher CRP levels than those in the survival group. Additionally, higher rates of antibiotic use (≥2 classes) and greater carbapenem exposure were observed. Among the isolates, CRAb accounted for 80.6%, ST2 accounted for 76.12%, and KL2/3/7/77/160 accounted for 65.67%. The predominant KL type was KL3, found in 19.4% of the isolates. All ST2 and KL2/3/7/77/160 isolates were CRAb. Among the isolates, 90.7% of the CRAb isolates coharbored *bla_OXA-23_* and *bla_OXA-66_*, while one coharbored *bla_NDM-1_* and *bla_OXA-23_*. Compared with non-ST2 and non KL2/3/7/77/160 infections, ST2 and KL2/3/7/77/160 infections had higher mortality rates (66.0% vs. 23.5%, P=0.002; 65.90% vs. 34.78%, P=0.015). Patients with ST2 and KL2/3/7/77/160 infections underwent more invasive procedures, received two or more antibiotics and carbapenem therapy before isolation, and had lower serum albumin levels. These isolates exhibited significantly higher resistance to antimicrobial agents. No significant differences in virulence phenotypes were observed between the two groups, except for biofilm formation between the ST2 and non-ST2 groups (P=0.002). However, these isolates harbored more virulence genes related to iron uptake and biofilm formation.”

The corrected section appears below:

“Results: The 30-day mortality rate of 67 patients with BSIs was 55.22%. Patients in the death group had significantly lower platelet counts and higher CRP levels than those in the survival group. Additionally, higher rates of antibiotic use (≥2 classes) and greater carbapenem exposure were observed. Among the isolates, CRAb accounted for 80.6%, ST2 accounted for 76.12%, and KL2/3/7/77/160 accounted for 65.67%. The predominant KL type was KL3, found in 19.4% of the isolates. All ST2 and KL2/3/7/77/160 isolates were CRAb. Among the isolates, 90.7% of the CRAb isolates coharbored *bla_OXA-23_* and *bla_OXA-66_*, while one coharbored *bla_NDM-1_* and *bla_OXA-23_*. Compared with non-ST2 and non KL2/3/7/77/160 infections, ST2 and KL2/3/7/77/160 infections had higher mortality rates (66.0% vs. 23.5%, P=0.002; 65.90% vs. 34.78%, P=0.015). Patients with ST2 and KL2/3/7/77/160 infections underwent more invasive procedures, received two or more antibiotics and carbapenem therapy before isolation, and had lower serum albumin levels. These isolates exhibited significantly higher resistance to antimicrobial agents. No significant differences in virulence phenotypes were observed between the two groups, except for biofilm formation between the ST2 and non-ST2 groups (P=0.035). However, these isolates harbored more virulence genes related to iron uptake and biofilm formation.”

The authors apologize for this error and state that this does not change the scientific conclusions of the article in any way. The original article has been updated.

